# Diabetes mellitus is a risk factor for prolonged SARS-CoV-2 viral shedding in lower respiratory tract samples of critically ill patients

**DOI:** 10.1007/s12020-020-02465-4

**Published:** 2020-09-01

**Authors:** Niccolò Buetti, Pierpaolo Trimboli, Timothy Mazzuchelli, Elia Lo Priore, Carlo Balmelli, Alexandra Trkola, Marco Conti, Gladys Martinetti, Luigia Elzi, Alessandro Ceschi, Vera Consonni, Adam Ogna, Valentina Forni-Ogna, Enos Bernasconi

**Affiliations:** 1 Ente Ospedaliero Cantonale, Regional Hospital Locarno, Locarno, Switzerland; 2 University of Paris, INSERM IAME, U1137, Team DeSCID, Paris, France; 3 Infection Control Program and World Health Organization Collaborating Centre on Patient Safety, University Hospitals and Faculty of Medicine, University of Geneva, Geneva, Switzerland; 4 Faculty of Biomedical Sciences, Università della Svizzera Italiana (USI), Lugano, Switzerland; 5 Clinic for Nuclear Medicine and Competence Center for Thyroid Diseases, Imaging Institute of Southern Switzerland, Ente Ospedaliero Cantonale, Bellinzona, Switzerland; 6 Ente Ospedaliero Cantonale, Infection Control Program, Ticino, Switzerland; 7 Ente Ospedaliero Cantonale, Division of Infectious Diseases, Regional Hospital Lugano, Lugano, Switzerland; 8 Institute of Medical Virology, University of Zurich, Zurich, Switzerland; 9 Laboratory of Microbiology EOLAB, Bellinzona, Switzerland; 10 Division Infectious Diseases, Regional Hospital Bellinzona, Bellinzona, Switzerland; 11 Division of Clinical Pharmacology and Toxicology, Institute of Pharmacological Sciences of Southern Switzerland, Ente Ospedaliero Cantonale, Lugano, Switzerland; 12 Department of Clinical Pharmacology and Toxicology, University Hospital Zurich and University of Zurich, Zurich, Switzerland

**Keywords:** Viral shedding, Infectivity, COVID-19, SARS-CoV-2, Intensive care unit, Type 2 diabetes mellitus

## Abstract

**Purpose:**

The length of time a critically ill coronavirus disease 2019 (COVID-19) patient remains infectious and should therefore be isolated remains unknown. This prospective study was undertaken in critically ill patients to evaluate the reliability of single negative real-time polymerase chain reaction (RT-PCR) in lower tracheal aspirates (LTA) in predicting a second negative test and to analyze clinical factors potentially influencing the viral shedding.

**Methods:**

From April 9, 2020 onwards, intubated COVID-19 patients treated in the intensive care unit were systematically evaluated for severe acute respiratory syndrome coronavirus 2 (SARS-CoV-2) by RT-PCR of nasopharyngeal swabs and LTA. The time to negativity was defined as the time between the onset of symptoms and the viral clearance in LTA. In order to identify risk factors for prolonged viral shedding, we used univariate and multivariate Cox proportional hazards models.

**Results:**

Forty-eight intubated SARS-CoV-2 patients were enrolled. Overall, we observed that the association of the first negative RT-PCR with a second negative result was 96.7%. Median viral shedding was 25 (IQR: 21.5–28) days since symptoms’ onset. In the univariate Cox model analysis, type 2 diabetes mellitus was associated with a prolonged viral RNA shedding (hazard ratio [HR]: 0.41, 95% CI: 0.06–3.11, *p* = 0.04). In the multivariate Cox model analysis, type 2 diabetes was associated with a prolonged viral RNA shedding (HR: 0.31, 95% CI: 0.11–0.89, *p* = 0.029).

**Conclusion:**

Intubated patients with type 2 diabetes mellitus may have prolonged SARS-CoV-2 shedding. In critically ill COVID-19 patients, one negative LTA should be sufficient to assess and exclude infectivity.

## Introduction

Treatment of critically ill coronavirus disease 2019 (COVID-19) patients requires strict isolation to limit nosocomial spread of severe acute respiratory syndrome coronavirus 2 (SARS-CoV-2). However, to date, the length of time an individual infected with SARS-CoV-2 remains infectious and needs to be isolated remains unknown, as viral shedding has been described to persist for an interval varying between 10 [1] and 60 days [2] after symptoms’ onset. Mild cases may have an earlier viral clearance (i.e., negative real-time polymerase chain reaction (RT-PCR) or cell cultures by day 10 post onset) [1, 3], whereas severe cases may have longer viral shedding [4, 5]. Most studies focused on nasopharyngeal or oropharyngeal swabs [3, 4, 6–9] and did not consider lower respiratory samples. Most importantly in this context, only one study (until June 2020) assessed the infectivity with cell culture for SARS-CoV-2 [1].

This prospective study was undertaken in critically ill patients to (1) evaluate the reliability of single negative RT-PCR in predicting a second negative test, (2) investigate the interval from symptoms’ onset to RT-PCR negativity, and (3) analyze clinical factors potentially influencing the latter.

## Methods

### Geographic and demographic context

**Fig. 1:**
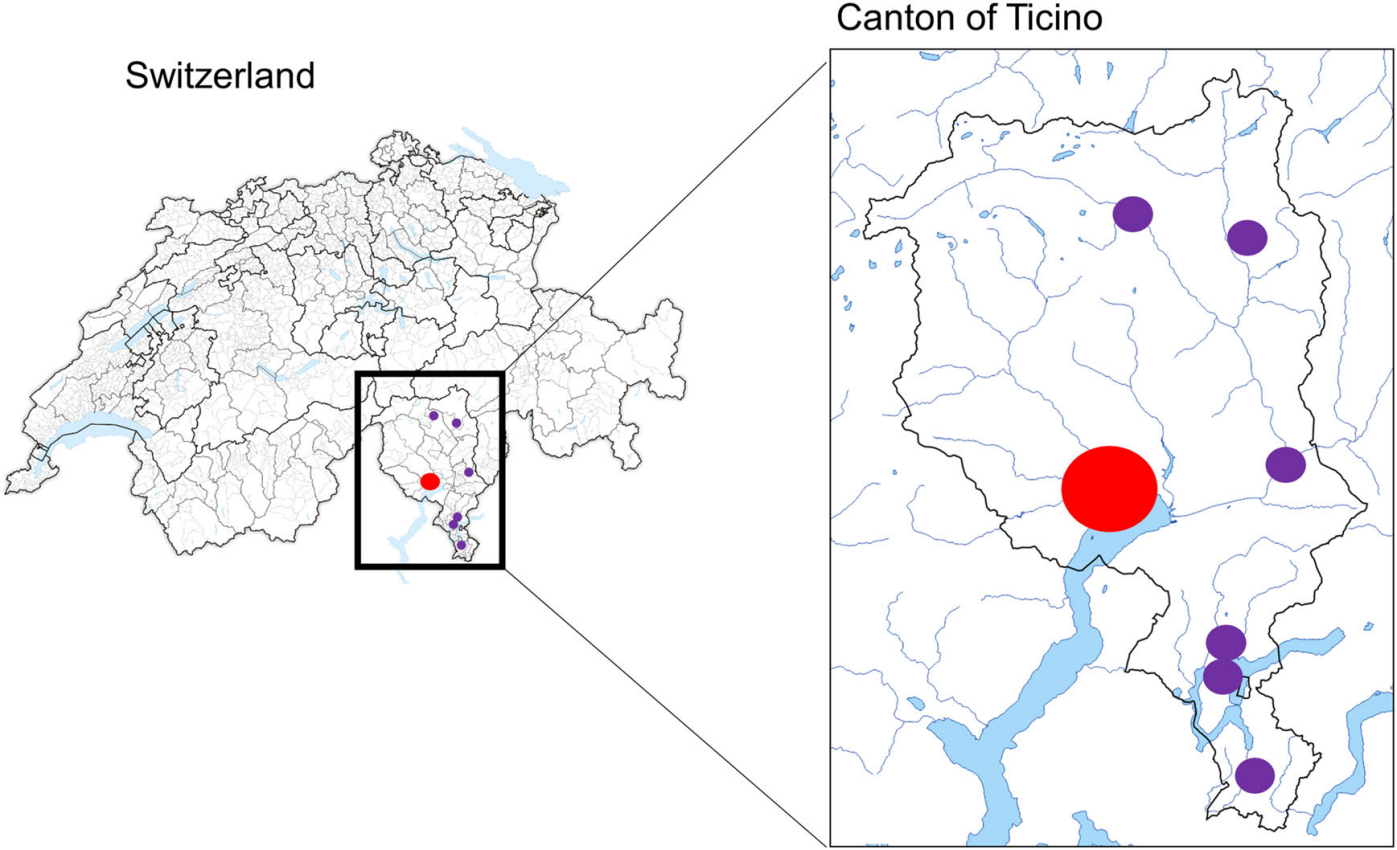
Geographic description of public health hospital network of Ticino, the Italian speaking canton in the Southern part of Switzerland. The different community hospitals are shown in the circles. The Locarno community hospital (SARS-CoV-2 dedicated center) is shown by the largest red circle

The study was performed at Locarno community hospital, a 250 beds facility entirely dedicated to COVID-19 patients during the SARS-CoV-2 pandemic, and is part of the public hospital network of southern Switzerland, serving an area of 350,000 inhabitants and being close to the SARS-CoV-2 outbreak epicenter of Northern Italy (Fig. 1). All data of SARS-CoV-2 patients were collected in a specific standardized institutional database.

### Institutional management of intubated SARS-Cov-2 patients

**Fig. 2:**
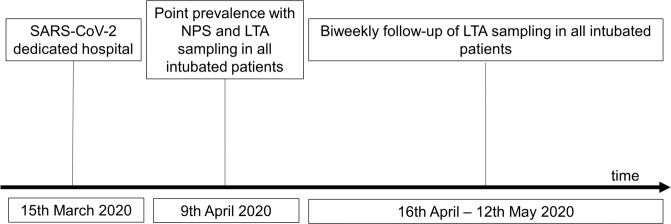
Timeline of RT-PCR evaluation according to the institutional management during the SARS-CoV-2 pandemic. NPS nasopharyngeal sample, LTA lower tracheal aspirate. Point prevalence: LTA and NPS were performed at 2 consecutive days in all intubated patients. During the follow-up NPS were performed only if an NPS was positive during the point prevalence assessment

From April 9, 2020 onwards, COVID-19 patients treated in the intensive care unit (ICU) were systematically evaluated for SARS-CoV-2 by RT-PCR of nasopharyngeal swabs (NPS) and lower tracheal aspirates (LTA). After their initial test, all patients underwent a biweekly LTA RT-PCR reevaluation until removal of the endotracheal tube or death or, according to the literature [10], until two consecutive negative RT-PCR results (Fig. 2). Furthermore, in selected cases and in order to prove the true negativity of a single negative RT-PCR, a viral culture was performed. In the included patients the results of NPS were also routinely collected.

### Patient selection and variables collected

All intubated ICU patients hospitalized at our institution between April 9 and May 12, 2020 were retrospectively reviewed. The following variables were routinely collected: demographic, clinical, laboratory, and microbiological data on SARS-CoV-2 patients, ICU-specific complications, and information on management. Mortality was assessed on May 20, 2020.

### Microbiological analyses: RT-PCR and viral cultures

Sampling was performed according to a designed protocol. Swab samples were immediately inserted into sterile tubes containing 3 mL UTM-RT^®^ viral transport medium (Copan, Brescia Italy), LTA were placed into sterile tubes. Both types of specimens were sent to the microbiology laboratory for sample processing and viral RNA extraction, which were performed on the same day. RT-PCR on NPS were performed using the commercial kit SARS-CoV-2 S Gene VIASURE Real Time PCR detection kit by CerTest BIOTEC on BD MAX Instrument (Becton Dickinson, New Jersey, USA). RNA extracted from LTA were amplified using a second PCR system based on the protocol published by Corman et al. [11] with primers and probes produced by TibMol Biol (Berlin, Germany). The viral load was indicated as cycle threshold (Ct) value of S gene of SARS-CoV-2 (VIASURE) and E- and RdRP gene, respectively. A positive and a negative control, as well as internal controls, was included in the assay, according to the manufacturer’s protocol. A Ct value of <40 was defined as positive for SARS-CoV-2 RNA and >40 was defined as negative. Samples with a Ct value between 37 and 40 were retested, at least twice.

For virus cultures, patient material was precleared by centrifugation and filtered before inoculation of Vero CCL81 cells with a dilution series of the patient material. Cells were observed daily for cytopathic effects (CPE) and upon detection of CPE or at latest by d7 supernatants were harvested and tested for the presence of SARS-CoV-2 by RT-qPCR.

### Outcome

The onset of symptoms was used as the starting time point for the viral clearance process. The date of the first negative detection of viral RNA (i.e., time to negativity or viral shedding) was defined as the end time point of viral clearance (see “Results” section).

### Statistical analyses

The time to negativity was described using descriptive statistics (median and IQR). In order to identify risk factors for prolonged viral shedding, we used univariate Cox proportional hazards models. To date, no study exhaustively investigated risk factors for prolonged viral shedding and, therefore, we performed multivariate Cox models including variables that showed *p* values < 0.20 in the univariate analysis. A hazard ratio (HR) of <1 indicated prolonged viral RNA shedding. In the Cox models, the proportionality of hazard risks was tested using Martingale residuals. The graphical of daily risk for prolonged viral shedding was illustrated using the hazard rate function from right-censored data using kernel-based methods [12]. All statistical analyses were performed with SAS (version 9.4).

## Results

### Demographic features

According to the study design and selection criteria, 48 intubated SARS-CoV-2 patients were enrolled. Overall, 161 RT-PCR tests of LTA were performed and patients underwent two to eight evaluations. The median time from onset of symptoms and the first sampling was 28 days (IQR: 23.5–32.5). Table 1 details the characteristics of the patients. Twelve patients (25%) patients died until May 20, 2020. At screening time, 31 patients (65%) showed same results between NPS and LTA, whereas in 15 patients (31%) LTA samples were positive and NPS were negative.

**Table 1:** Patients’ characteristics

Feature	
Age	
Median (IQR)	66.5 [60; 71]
Sex	
Female *n* (%)	11 (22.9)
Comorbidities, *n* (%)	38 (79.2)
Cardiovascular, *n* (%)	14 (29.2)
Chronic respiratory failure, *n* (%)	6 (12.5)
Chronic renal insufficiency, *n* (%)	3 (6.3)
Active solid or hematologic neoplasia, *n* (%)	4 (8.3)
Type 2 diabetes mellitus, *n* (%)	13 (27.1)
Hypertension, *n* (%)	24 (50)
BMI median (IQR), *n* (%)	29 [25; 33]
Chronic immunosuppression, *n* (%)	1 (2.1)
Symptoms at admission	
Fever, *n* (%)	44 (91.7)
Cough, *n* (%)	35 (72.9)
Fatigue, *n* (%)	29 (60.4)
Diarrhea, *n* (%)	11 (22.9)
Vomiting, *n* (%)	5 (10.4)
Antivirals	27 (56.3)
Lopinavir/ritonavir, *n* (%)	20 (41.7)
Hydroxychloroquine, *n* (%)	21 (43.8)
Remdesivir, *n* (%)	7 (14.6)
Tocilizumab, *n* (%)	5 (10.4)
Laboratory at admission	
Lymphocyte 10^9^/L, median (IQR)	0.7 [0.6; 0.9]
Thrombocyte 10^9^/L, median (IQR)	166 [148.5; 186.5]
CRP mg/L, median (IQR)	95.5 [53; 193.5]
Creatinine umol/L, median (IQR)	95.5 [81.5; 123]
Lymphopenia duration, days median (IQR)	24.5 [18.5; 32]
Reasons for ICU admission	
Acute respiratory failure, *n* (%)	46 (95.8)
Sepsis or septic shock, *n* (%)	1 (2.1)
Cardiac failure, *n* (%)	1 (2.1)
SAPS II at ICU admission	
Median (IQR)	47 [39.5; 58.5]
Severe ARDS, *n* (%)	36 (75)
Noninfectious complications during ICU stay	
Thrombosis, *n* (%)	25 (52.1)
Acute kidney injury, *n* (%)	22 (45.8)
Infectious complications during ICU stay	
VAP, *n* (%)	33 (68.8)
UTI, *n* (%)	10 (20.8)
Candidemia, *n* (%)	5 (10.4)
Corticosteroids during ICU stay, *n* (%)	25 (52.1)
Time to negativity, days median (IQR)	25 [21.5; 28]
Mortality, *n* (%)	12 (25)

*IQR* interquartile range, *BMI* body mass index, *ICU* intensive care unit, *SAPS* Simplified Acute Physiology Score, *VAP* ventilator-associated pneumonia, *UTI* urinary tract infection

### Evaluation of single negative RT-PCR as predictor of second negative test

In total, 31 patients with one negative RT-PCR in LTA during their follow-up and 28 (90.3%) had a second negative test within the following 3 days. Two of the remaining three cases had a negative test within 1 week. In one case, the subsequent RT-PCR was positive with 37-Ct. Among these 31 patients, data on the viral culture of LTA samples were available in four cases at their time of first negative RT-PCR and all these patients had both negative RT-PCR and culture results. Overall, we observed that the association of the first negative RT-PCR with a second negative result was 96.7%.

### Analysis of viral shedding

Based on the above results, we are confident that a single negative RT-PCR in LTA can serve as a proof of negativity. Median viral shedding was 25 (IQR 21.5–28) days since symptoms’ onset (Table 1).

### Risk factors for prolonged viral shedding

**Fig. 3:**
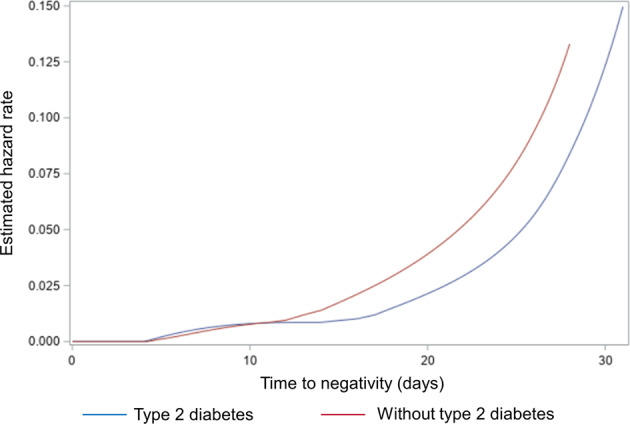
Estimated hazard rate for type 2 diabetes mellitus and time to negativity based on univariate Cox model

In the univariate Cox model analysis, type 2 diabetes mellitus was associated with a prolonged viral RNA shedding (HR: 0.41, 95% CI: 0.06–3.11, *p* = 0.04, Table 2 and Fig. 3).

**Table 2:** Univariate and multivariate Cox models for time to negativity

	Univariate model			Multivariate model^a^		
	HR	95% CI	*p* value	HR	95% CI	*p* value
Age	1.04	0.99–1.09	0.14	1.05	0.995–1.12	0.071
Sex, female	1.78	0.81–3.93	0.15	1.35	0.58–3.12	0.490
Comorbidities	0.64	0.27–1.53	0.32			
Cardiovascular disease	0.85	0.4–1.82	0.68			
Type 2 diabetes mellitus	0.41	0.17–0.97	0.04	0.313	0.11–0.89	0.029
Hypertension	1.23	0.59–2.56	0.58			
BMI	1.03	0.97–1.09	0.38			
Chronic respiratory failure	0.69	0.25–1.86	0.46			
Chronic renal failure	0.42	0.06–3.11	0.40			
Solid or hematologic neoplasia	2.08	0.62–6.99	0.24			
Lymphopenia duration	0.98	0.94–1.01	0.23			
SAPS II at ICU admission	1.01	0.99–1.03	0.42			
ARDS, severe	0.59	0.25–1.39	0.23			
Thrombosis during ICU stay	1.33	0.64–2.77	0.44			
AKI during ICU stay	0.60	0.29–1.25	0.17	1.09	0.48–2.52	0.84
VAP during ICU stay	0.80	0.38–1.66	0.54			
UTI during ICU stay	1.10	0.47–2.59	0.82			
Colitis during ICU stay	2.56	0.33–19.6	0.37			
Corticosteroids during ICU stay	1.10	0.54–2.24	0.80			
Antivirals	0.63	0.30–1.33	0.23			
Tocilizumab	1.40	0.41–4.72	0.59			

The proportionality hazard was assessed using Martingale residuals and was respected for diabetes mellitus (*p* = 0.38)

*HR* hazard ratio, *CI* confidence interval, *BMI* body mass index, *SAPS* Simplified Acute Physiology Score, *ICU* intensive care unit, *VAP* ventilator-associated pneumonia, *UTI* urinary tract infection, *AKI* acute kidney insufficiency

^a^Variables included in the multivariate analysis were age, sex, type 2 diabetes mellitus, and AKI during ICU stay

On the contrary, lymphopenia duration (HR: 0.98, 95% CI: 0.94–1.01, *p* = 0.23), ventilator-associated pneumonia (VAP) during ICU stay (HR: 1.33, 95% CI: 0.38–1.66, *p* = 0.54), severe ARDS (HR: 0.59, 95% CI: 0.25–1.39, *p* = 0.23), and antivirals (HR: 0.63, 95% CI: 0.30–1.33, *p* = 0.23) did not significantly influence the duration of viral shedding. Also in the multivariate Cox model analysis type 2 diabetes mellitus was associated with a prolonged viral RNA shedding (HR: 0.31, 95% CI: 0.11–0.89, *p* = 0.029, Table 2), and similar results were observed in a sensitivity analysis defining negativity using two negative LTA samples (HR: 0.23, 95% CI: 0.07–0.69, *p* = 0.0089, Table 3). Of note, the mortality was higher among type 2 diabetes patients (54 versus 14%, *p* < 0.01).

**Table 3:** Multivariate Cox analysis using two negative LTA samples for negativity

	Multivariate analysis		
	HR	95% CI	*p* value
Age	1.05	0.99–1.105	0.080
Sex, female	1.52	0.64–3.63	0.35
Type 2 diabetes mellitus	0.23	0.07–0.69	0.0089
AKI during ICU stay	1.41	0.59–3.36	0.44

*HR* hazard ratio, *CI* confidence interval, *ICU* intensive care unit, *AKI* acute kidney insufficiency, *LTA* lower tracheal aspirate

## Discussion

This study highlighted two important points concerning viral shedding in critically ill COVID-19 patients: (1) type 2 diabetes mellitus is associated with prolonged SARS-CoV-2 shedding; (2) a first negative LTA sample is predictive for a second negative sample.

Type 2 diabetes patients are at the highest risk for complications from COVID-19 infection and several authors described the relationship between COVID-19 and diabetes [13, 14]. We showed that type 2 diabetes mellitus was identified as a risk factor for prolonged SARS-CoV-2 shedding. We suggest the following possible pathophysiological explanations: (1) the innate immune system is the first line of defense against SARS-CoV-2, and may be compromised in patients with uncontrolled type 2 diabetes [15, 16]; (2) patients with type 2 diabetes mellitus may be more susceptible to an inflammatory cytokine storm eventually leading to rapid deterioration of COVID-19 [17] and to reduced control of viral shedding; (3) type 2 diabetes mellitus reduced the expression of angiotensin-converting enzyme 2 which may play a potent anti-inflammatory and antioxidant role in the lung and, probably, may prolong viral shedding [18]. Therefore, critically ill people with type 2 diabetes who are infected with COVID-19 may require a prolonged infection prevention measures during the hospitalization. Interestingly, corticosteroid therapy during ICU stay, VAP, antiviral therapy, lymphopenia, and severe ARDS did not significantly influence the duration of viral shedding in our study population.

The ECDC recommends two consecutive negative RT-PCR tests from respiratory specimens at 24 h interval at least 8 days after symptoms onset to discontinue isolation precautions in hospitalized COVID-19 patients [10]. Similar recommendations were issued by several institutions in other continents [5, 19]. Specimens were usually collected in the upper respiratory tract, and data on lower respiratory samples are rarely considered. Viral shedding from upper respiratory tract appeared to be higher soon after symptoms’ onset; however, during the course of disease, viral shedding is predominantly located in the lower respiratory tract [1]. Using lower respiratory tract samples, we showed that one negative RT-PCR LTA sample is sufficient to confirm the absence of viral shedding in critically ill intubated COVID-19 patients, and the results of RT-PCR were also confirmed in selected patients by viral cell cultures. These findings highlight the importance of virological monitoring of LTA samples in critically ill patients to decide when isolation precautions for the prevention of respiratory infections could be discontinued, thus making an early transfer to general wards possible, and encouraging an early rehabilitation program that may accelerate the discharge at home and the reintegration into society.

Our study has several limitations. First, we performed an observational study using surveillance data. Second, LTA samples were not collected on a daily basis. Third, viral cell cultures were not routinely performed during the study. It is therefore conceivable that the results on viral shedding might have been different if this method would have been used systematically. Third, due to the relatively small sample size, the statistical analyses should be interpreted with caution. Finally, we did not collect further LTA samples after two negative results.

Intubated patients with type 2 diabetes mellitus may have prolonged SARS-CoV-2 shedding. In critically ill COVID-19 patients, one negative LTA should be sufficient to assess and exclude infectivity.

## Data Availability

The data sets used and/or analyzed during the current study are available from the corresponding author on reasonable request.
